# Antibiotic Persistence as a Metabolic Adaptation: Stress, Metabolism, the Host, and New Directions

**DOI:** 10.3390/ph11010014

**Published:** 2018-02-01

**Authors:** Damien J. Cabral, Jenna I. Wurster, Peter Belenky

**Affiliations:** Department of Molecular Microbiology and Immunology, Division of Biology and Medicine, Brown University, Providence, RI 02912, USA; damien_cabral@brown.edu (D.J.C.); jenna_wurster@brown.edu (J.I.W.)

**Keywords:** persistence, tolerance, metabolism, biofilms, next-generation sequencing

## Abstract

Persistence is a phenomenon during which a small fraction of a total bacterial population survives treatment with high concentrations of antibiotics for an extended period of time. In conjunction with biofilms, antibiotic persisters represent a major cause of recalcitrant and recurring infections, resulting in significant morbidity and mortality. In this review, we discuss the clinical significance of persister cells and the central role of bacterial metabolism in their formation, specifically with respect to carbon catabolite repression, sugar metabolism, and growth regulation. Additionally, we will examine persister formation as an evolutionary strategy used to tolerate extended periods of stress and discuss some of the response mechanisms implicated in their formation. To date, the vast majority of the mechanistic research examining persistence has been conducted in artificial in vitro environments that are unlikely to be representative of host conditions. Throughout this review, we contextualize the existing body of literature by discussing how in vivo conditions may create ecological niches that facilitate the development of persistence. Lastly, we identify how the development of next-generation sequencing and other “big data” tools may enable researchers to examine persistence mechanisms within the host to expand our understanding of their clinical importance.

## 1. Introduction

The discovery of antibiotics and their widespread use in the 20th century represent a significant milestone in human history. Commercial antibiotics have saved innumerable lives, but their efficacy has declined at an alarming rate due to the spread of antibiotic resistance. Within a decade of the first major utilization of penicillin therapy in soldiers during World War II [1], penicillin resistance became a significant clinical burden and signaled the beginning of an “arms race” between pathogenic bacteria and pharmaceutical development [2]. In addition to resistance, physicians such as Joseph Bigger were vexed by a concerning phenomenon; although penicillin was frequently and successfully used to treat *Staphylococcal* wound infections, therapies often failed to completely sterilize the infection site, ultimately resulting in severe infection relapse and mortality [3]. Bigger coined the term “persisters” to describe a minority subpopulation of bacterial cells that could survive antibiotic challenge in the absence of resistance [3,4]. Here, we define persisters as a small fraction of a total bacterial population that can survive long-term treatment with high concentrations of antibiotics. However, unlike resistant bacteria, most of these cells regain sensitivity after regrowth and new treatment typically results in the same small surviving fraction. Additionally, the phenomenon of tolerance is closely related to and often confused with persistence. Tolerance also enables bacterial cells to survive exposure to lethal concentrations of antibiotics; however, unlike persisters, tolerant cells make up a larger portion of the population and they are only temporarily protected from antibiotic exposure.

Over the last 60 years, an expansive body of work has focused on characterizing the genetic determinants, molecular mechanisms, and epidemiology of antibiotic resistance. Although the breadth of research on antibiotic persistence is less robust, the past decade has seen burgeoning interest in persistence as a cause of clinical therapeutic failure [1]. In recent years, the defining characteristics of persisters and their formation have been codified in primary literature and multiple reviews [4,5,6,7,8,9]. In this review, we aim to link work from the distinct fields of systems biology and in vivo clinical microbiology. Although these fields have been operating somewhat independently, we feel they are intrinsically related and together can help to decipher the heterogeneous phenomenon of antibiotic persistence. We will discuss antibiotic persistence as it relates to bacterial metabolism, specifically focusing on how carbon catabolite repression, sugar metabolism, and growth regulation are involved in persister formation. We will contextualize these findings by discussing how in vivo conditions create ecological niches that facilitate persistence development. Finally, we will discuss how persister formation represents a unique evolutionary strategy to combat antibiotic stress as well as some of the response mechanisms implicated in persister formation.

As we discuss this previous research, it is important to consider that a majority of mechanistic studies on persisters have been conducted under artificial conditions in vitro. In reality, antibiotics act on and induce persisters in complex polymicrobial communities that are themselves profoundly impacted by the host environment. Thus, the insight generated from this work may not be fully biologically relevant or clinically applicable. However, the development of new tools based on next-generation sequencing and “big data” analysis may allow us to study persistence and persistence-related processes in the host. Throughout this review, we will identify applications where these tools can be utilized to expand our understanding.

## 2. Persistence as an Evolutionary Adaptation

The term persistence describes the ability of a bacterial subpopulation to survive antibiotic exposure due to non-heritable phenotypic variation that is distinct from the mechanisms that generate resistance [1]. Persisters represent a small fraction of the total cells, but their survival allows the population to survive times of high antibiotic exposure [4]. After stress subsides, persisters revert to an antibiotic-sensitive state, reinitiate growth, and repopulate the local environment. In fact, post-treatment sensitization towards antibiotics is a definitive characteristic of persister cells [9]. This phenomenon is akin to ecological succession, where antibiotic pressure represents a bottleneck event and persisters are the first to subsequently utilize available nutrients and environmental niches. Like a wildfire that decimates a forest, antibiotic exposure wipes out 99 percent of a susceptible community while persister cells survive as a result of their transient antibiotic tolerance. As the sole survivors of antibiotic exposure, these persister cells then function as the pioneer “species” in a now-vacant ecological niche and subsequently lose their tolerant phenotype as they repopulate and grow towards a steady state community. In this manner, persistence can be viewed as an evolutionary strategy by which a population assures its survival through a few key members.

As an adaptive trait, persistence is heterogeneous and emerges via multiple mechanisms. Persistence has thus been categorized into subtypes for clarification. First, time-dependent persistence is contingent on growth rate reductions within the persister subpopulation that reduce antibiotic uptake and target availability [10]. Time-dependent persistence can be further subdivided into Type I and Type II persistence, where Type I is triggered by a reduced lag time and Type II is triggered by growth rate reduction [4,10]. Second, dose-dependent persistence is an adaptive response in which transient overexpression of efflux pumps and stress response pathways facilitate survival during antibiotic challenge [4,10,11]. The PASH (Persistence As Stuff Happens) model has recently gained popularity and suggests that both time- and dose-dependent persistence are the result of stochastic errors in metabolism, cell division, and stress responses and is thus analogous to spontaneous mutations observed in antibiotic resistance [12]. PASH suggests that persistence is a form of bet-hedging or adaptive behavior in which a small subset of the population exhibits randomized phenotypic variation. The utilization of toxin-antitoxin modules serves as one example of this bet-hedging strategy. Perhaps the best characterized of these systems is the *hipAB* module in *Escherichia coli* [13]. In this case, *E. coli* enter into a dormant state once the levels of the *hipA* toxin exceed a certain threshold [13]. Overexpression of *hipA* increases the tolerance of *E. coli* to bactericidal antibiotics [14]. However, these toxin levels fluctuate within a population in the absence of antibiotics, suggesting that they may represent a generalized response that allows bacterial populations to survive sudden stress [13,14,15]. Compared to antibiotic resistance, this randomization confers a selective advantage with a significantly diminished fitness cost and reduced need for compensatory adaptations [16].

Vogwill et al. recently aimed to identify whether persistence and resistance represent complementary, albeit divergent, survival strategies that bacteria have co-opted to survive antibiotic and environmental stressors [17]. After challenging various *Pseudomonas* species with ciprofloxacin and rifampin, they found that persistence and resistance generation were mechanistically unrelated but positively correlated, suggesting that they represent complementary, rather than competitive, evolutionary strategies [17]. Persistence is a plastic trait, while resistance is genetically encoded. If antibiotic exposure is constant, there would be no need for the evolution of plastic traits and selection would favor resistance. However, if antibiotic exposure is transient, selection should favor phenotypic plasticity due to the higher fitness costs of resistance relative to persistence. By maintaining a variant subpopulation, the bacterial population as a whole ensures its survival in times of transient stress [17].

**Figure 1:**
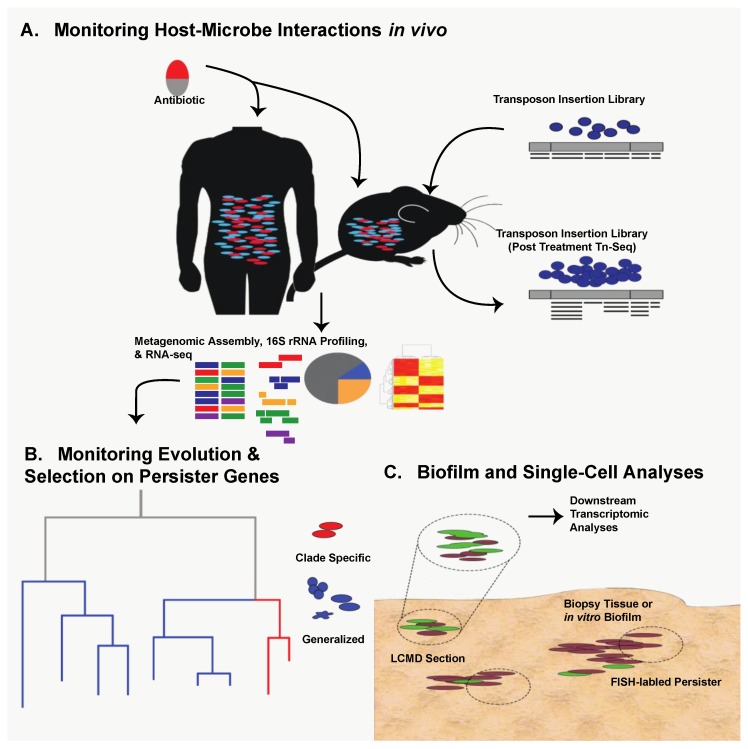
Utilization of next-generation technologies for studying persister cells. (**A**) Both human patients and murine models provide an opportunity to study in vivo persister formation via 16S rRNA profiling, community metagenomics, and RNA-Seq of the intestinal flora following antibiotic exposure. Single-organism persister formation can be studied in vivo through animal infection with high-density transposon insertion libraries and Tn-Seq. (**B**) Metagenomic and RNA-Seq data can be used to study selective pressure on persister genes in either closely related or divergent taxa. This can be done in retrospective clinical cohort groupings or in animal model infections over the course of antibiotic therapy, identifying how certain therapeutic regimens can select for the expression of specific (red) or ubiquitous (blue) persister elements. (**C**) Persisters can be studied from either in vitro-generated biofilms or patient biopsy-derived biofilms. Persister-specific Fluorescence In-Situ Hybridization (FISH) labeling can allow for visualization and study of persisters within the 3D context of the biofilm, and laser-capture microdissection (LCMD) sectioning can facilitate labeled cell extraction for downstream transcriptomic analyses.

Thanks to technological expansion in genomics during the last decade, persister research can capitalize on next-generation methods used in virulence and antibiotic resistance studies. Transposon-sequencing (Tn-seq) is an attractive, massively parallel means of identifying persister-associated gene targets in vivo under various selective conditions [18,19,20] (Figure 1A). Tn-seq has the potential to confirm the importance of known persister genes under specific stress conditions as well as identify novel persistence mechanisms in the host. In addition to new in vivo work, retrospective genomic studies can be used to identify known persister genes with varying amounts of selective pressure. Specifically, it may be beneficial to analyze clinical isolates of common pathogenic bacteria taken over the last sixty years of antimicrobial availability to ascertain evolutionary pressure and conservation of key persister genes [17] (Figure 1B).

## 3. Biofilms Can Promote Antibiotic Persistence in Clinical Settings

While persister development is an adaptive strategy at an individual level, bacteria can also exhibit community-wide adaptations to survive antibiotic challenge. Committing to a biofilm community structure can aid in bacterial fitness and promote persister development, particularly if the biofilm is slow-growing in nature [21]. Biofilms are an amalgam of one or more bacterial species that colonize and adhere to physical surfaces in a density-dependent manner. The biofilm creates heterogeneous gradients in signaling molecules, nutrients, and environmental exposures that generate diverse micro-niches [1]. Biofilm formation is found ubiquitously across microbial phyla, and facilitates colonization of both abiotic and biotic surfaces with relative ease [22].

Biofilms are characteristically stress-resilient, and they are a great example of how population size and fitness are positively correlated through the Allee effect [23]. The Allee effect describes scenarios in which biological characteristics correlate the population density of a given ecosystem with the fitness of individual species or the population within that ecosystem [23]. In microbial ecology, biofilms increase the total population density irrespective of whether they are mono- or polymicrobial. As the population becomes stabilized by density, intraspecies variation and thus fitness drastically increase due to cooperative interactions and reduced genetic drift [24,25]. The biofilm as a total population exhibits antibiotic tolerance, and increased cooperative interactions within the population might generate conditions that increase persister cell formation.

Clinically, biofilms are associated with antibiotic recalcitrance, infection recurrence, and persister formation [1]. Biofilm formation has been documented in both Gram-positive and Gram-negative pathogens and is clinically significant in various infection types, ranging from skin and soft tissue infections (SSTI), implanted device infections, urinary tract infections, endocarditis, otitis media, and more [12,22,26,27,28]. Approximately 50 percent of all nosocomial infections originate from implanted medical devices such as prosthetic joints, catheters, and prosthetic heart valves, all of which provide abiotic surfaces for the development of biofilms [12]. In patients, tissue location and biofilm progression can result in varied antibiotic exposure even in the presence of clinically appropriate dosing [16]. Ultimately, this results in bacterial exposure to sub-inhibitory antibiotic concentrations, which can promote persister development [16,29,30,31].

Biofilm formation appears to confer significant fitness advantages to pathogenic bacteria. As an environment subject to ecological drivers, biofilms promote intraspecies variation that encourages persister development. Lee et al. have suggested that heterogeneity promotes antibiotic tolerance through the altruistic behavior of a few variant subpopulations within the biofilm [32]. This “bacterial charity” is analogous to kin selection, where a subset of cells obtains resistance- or persistence-conferring capacity and provides protection to others. Lee found that mutations in indole production were directly correlated to charity events in polymicrobial biofilms. By challenging *E. coli* strains to increasing concentrations of fluoroquinolones, they found that a highly resistant and high indole-producing subpopulation triggered overall biofilm tolerance via indole signaling [32]. Biofilms are ultimately important to the study of persister formation because they represent the endogenous ecological structures that many bacteria will adopt within a host [33].

Persister cells are implicated as a causative agent in a multitude of biofilm-related recurrent infections including urinary tract infections, where sub-inhibitory antibiotic concentrations promote persister development and multi-drug tolerance [26,34,35]. In otitis media (OM), a commonly chronic or recurrent infection, the formation of a polymicrobial biofilm is initiated by opportunistic members of the nasopharyngeal microbiota that migrate towards the inner ear and trigger infection [27]. In OM, the role of cooperative intraspecies interactions has been well documented. In polymicrobial biofilms, cooperation promotes multi-drug tolerance by persister cells [27]. Specifically, *Moraxella catarrhalis* provides passive β-lactam protection to *Streptococcus pneumoniae* and non-typable *Haemophilus influenzae* (NTHi), and in turn they provide tolerance towards fluoroquinolones by promoting *M. catarrhalis* persister cell formation [27]. Here, as in periprosthetic joint implant infections, biofilm formation functions as a vehicle for persister cell development [36]. In *Staphylococcal* infections, recurrent SSTIs have been associated with biofilms in response to prolonged antibiotic exposure, including last-line therapies such as vancomycin [37]. In catheter-related bloodstream infections, there is an effective relapse rate of approximately 20 percent, due to surviving persister populations within catheter-adhered biofilms [28]. Even extended antibiotic therapy at 1000-fold inhibitory concentrations is insufficient to eliminate the biofilm [28]. Thus, the theme of a biofilm functioning as an environment that promotes tolerant infections and persister cell development is prominent in clinical settings.

The question then becomes how can we study persister formation in host-related biofilms? As with persister gene evolution, Tn-seq is an attractive option in which a host-related biofilm infection model can be established with a high-density transposon insertion library (Figure 1A). Alternatively, persister-specific Fluorescence In-Situ Hybridization (FISH) could be used to isolate biofilms from in vivo contexts and identify persisters in their native environment by quantifying expression of persister elements such as toxin-antitoxin systems [38,39]. Laser-capture microdissection (LCMD) could then be coupled with transcriptomic analysis to isolate specific, persister-containing fragments of the biofilm, assess their transcriptional activity, and decouple it from culture-specific or in vitro-specific variation (Figure 1C). The great strength of LCMD coupled with FISH is that it allows the transcriptional analysis of populations enriched for persisters. A similar approach could and has been taken to analyze transcriptional response of persisters in liquid culture through the use of flow cytometry sorting [40,41]. While these approaches enrich for persisters, clear challenges related to intrinsically low abundance of persisters still remain. However, as single-cell sequencing technologies advance, many of these challenges can be effectively solved by enabling analysis of rare persister cells [42,43,44,45,46,47,48]. The key to each of these approaches is that they allow monitoring of persister biology in biofilms generated within the host, providing additional translational impact.

## 4. Growth, Metabolism, and ATP Production

The formation of biofilms and persister cells represent two interrelated yet phenotypically distinct strategies utilized by bacteria to tolerate antibiotic treatment. Despite their differences, however, growth rate and the underlying metabolic state are crucial determinants of the antibiotic tolerance displayed by both biofilm and persister cells. As a complex ecological environment, biofilms exhibit heterogeneity in their population structure and metabolic activity. Cells proximal to the center of the biofilm can exhibit marked dormancy relative to cells in the periphery [49]. As a result, antibiotic efficacy is highest at the air interface, where metabolic activity is highest [49]. Additionally, the growth rate of biofilm cells has been shown to be a major determinant of antibiotic susceptibility in both *Pseudomonas aeruginosa* and *E. coli* [50,51]. Similar dynamics have also been observed in non-biofilm persister cells. Tolerance and persistence are closely associated with the rate and phase of bacterial growth [11,52,53,54,55]. For example, slow growth rates have been shown to permit stable tolerant phenotypes in *E. coli* [56], and both *P. aeruginosa* and *S. aureus* display an increase in persister formation in mid-exponential and stationary phase while remaining unchanged in early exponential phase [14]. Conversely, maintaining bacterial cultures in early exponential phase has been found to completely eliminate persisters [7,14]. Furthermore, *E. coli* has demonstrated an ability to modulate its lag time to match the duration of antibiotic exposure when subjected to repeated treatments [57]. Therefore, modulation of growth rate appears to be an adaptive and transient response to antibiotic exposure.

A major contributing factor to variations in growth rate is nutrient availability, with nutrient limitation having long been known to induce persistence. Glucose deprivation has been shown to increase the formation of persisters and increase biofilm tolerance to fluoroquinolone and β-lactam treatment [58,59]. Conversely, stationary phase *E. coli* cells can be sensitized to ciprofloxacin by supplementing oxygen and carbon sources [55]. Furthermore, *E. coli* grown in minimal media with limited glucose availability have higher expression of the efflux pump *acrB*, suggesting that sugar metabolism may have wide-ranging effects that include drug efflux [60]. It appears that amino acid deprivation is prerequisite for tolerance; however, deprivation of glucose in addition to amino acids produces bacteria that are highly tolerant of β-lactams, fluoroquinolones, and aminoglycosides [56].

Long-term starvation of *Mycobacterium tuberculosis* reduces susceptibility to rifampicin, isoniazid, and metronidazole and induces shifts in the expression of central metabolic pathways such as amino acid biosynthesis, energy metabolism, and lipid biosynthesis [53]. Most notably, starvation down-regulates the expression of many glycolysis and TCA cycle enzymes. Additionally, the NADH dehydrogenase operon and most of the ATP synthase complex, both of which contribute to the production of ATP, are dramatically down-regulated [53]. Conversely, starvation induced a significant up-regulation of the fumarate reductase gene *frdA*, which is a component of a complex which serves as an anaerobic electron transport chain in similar bacteria [53]. These changes allow *M. tuberculosis* to enter into a tolerant state by decreasing growth rate while maintaining viability [53]. Taken together, these findings further demonstrate that phenotypic plasticity in bacteria is critical to surviving antibiotic exposure events.

Bacteria may also reduce their metabolic flux and enter a persistent state through the utilization of the glyoxylate shunt. The glyoxylate shunt is a variant of the TCA cycle that enables net carbon assimilation by bypassing steps that generate carbon dioxide [61,62,63]. In *M. tuberculosis*, treatment with three distinct antibiotics (rifampicin, isoniazid, and streptomycin) is known to induce the expression of isocitrate lyase (*icl*), a component of the glyoxylate shunt that converts isocitrate to glyoxylate and succinate [11,63]. Furthermore, deletion of *icl* dramatically increases the susceptibility of *M. tuberculosis* to those drugs [11]. While nutrient starvation decreases the expression of most metabolic genes in *M. tuberculosis*, it has little effect on genes within the glyoxylate shunt, such as *icl* [53]. Utilization of the glyoxylate shunt decreases flux through the TCA cycle and reduces NADH and ATP production [11,54]. As a result, usage of the glyoxylate shunt is thought to result in reduced levels of reactive oxygen species (ROS), which may contribute to its protective effect [11,52,54]. Similar effects were seen in response to aluminum toxicity in *Pseudomonas fluorescens*, suggesting that metabolic tolerance mechanisms are utilized in other types of stress responses [54]. However, defects in the glyoxylate shunt have been shown to increase biofilm formation and tolerance of oxidative stress in *P. aeruginosa*, suggesting that this strategy is not universally employed, even amongst closely related bacteria [64].

Another stark example of metabolic modulation and persistence development is the phosphate metabolism gene *phoU* [65]. *E. coli* mutants lacking *phoU* are unable to resume growth following β-lactam exposure and are more susceptible to numerous antibiotics and stress conditions. While wild type persisters remain unsusceptible to all antibiotics tested, *phoU* mutants that survive initial antibiotic perturbation remain susceptible to β-lactams. Furthermore, loss of *phoU* sensitizes stationary phase *E. coli* to ampicillin, which requires active growth for effective killing of wild type cells [65]. Cells lacking *phoU* upregulate genes involved in energy production; for this reason, it has been suggested that *phoU* regulates persistence by reducing the expression of metabolic genes in response to stressors such as nutrient limitation or antibiotic exposure [65].

While persister cells, by definition, are not growing during antibiotic challenge, they can and do originate from actively dividing bacteria. Using fluorescent reporters for growth and metabolism, it was estimated that persisters constitute approximately 1 percent of stationary phase cells within an exponentially growing *E. coli* culture [66]. Within that same culture, only 0.01 percent of persisters originated from actively growing cells. However, because of the high prevalence of growing cells in an exponential culture, as many as 20 percent of persisters may originate from active cells [66]. Within the growing populations, decreased reductase activity was found to be closely associated with persister formation. In fact, growing cells with low reductase activity were 40 times more likely to become persisters [66]. Other studies have shown that bacterial cells with lower rates of protein synthesis were more likely to be persisters [31]. Interestingly, the gene expression profile for these cells more closely resembled exponential- rather than stationary-phase cells. Though energy metabolism was decreased in general, these cells also had increased expression of toxin-antitoxin systems [31]. Therefore, this suggests that although decreased metabolism does greatly increase the likelihood of persister formation, it is not sufficient to explain the phenotype [31,66].

It is clear that bacterial metabolic state is a major determinant of persister and biofilm formation in vitro. However, the metabolic conditions experienced in vitro are likely to differ dramatically from those encountered within the host. Therefore, it is likely that host metabolism plays a major role in bacterial functional potential [67]. Within the microbiome, metagenomics and metatranscriptomics can be utilized to profile the prevalence and expression of well-known tolerance and persistence genes in a polymicrobial community (Figure 1A). However, such analyses will not exclusively profile persister cells due to their rarity but may identify factors that allow populations to survive antibiotic treatment and promote persistence.

## 5. Carbon Catabolite Repression Systems Coordinate Antibiotic Persistence and Tolerance

Pathogenic microbes are heterotrophic and rely on a variety of carbon sources for growth [68]. The ability to sense and efficiently utilize a diverse pool of carbon sources, which increases nutritional fitness, is contingent upon highly coordinated metabolite sensing coupled with rapid and appropriate responses. Bacterial growth and metabolism are intricately linked to the availability of carbon sources and cellular responses to this availability. Thus, persister formation is also closely linked to carbon flux within the cell.

Perhaps one of the best described and most conserved metabolite response systems is the carbon catabolite repression (CCR) system. CCR is a global regulatory mechanism by which utilization of secondary carbon sources is dampened in the presence of preferred carbon sources such as glucose [69]. In Gram-negative species, CCR is activated by transcriptional repression of a pro-catabolic cyclic-AMP-CRP protein complex. In Gram-positive species, CCR is negatively regulated. Environmental glucose triggers phosphorylation of the histidine protein (HPr), which complexes with a pleiotropic transcription factor, carbon catabolite protein A (CcpA). This heterotropic complex binds to responsive DNA elements, thereby repressing catabolic gene expression [69]. CcpA has been demonstrated to regulate a massive proportion of glucose-responsive genes, almost 80 percent in *Bacillus subtilis*, and carbon sources have been implicated in *E. coli* persister formation, hinting at a possible connection between CCR and persisters [70].

Nutrient transitions, starvation, and the CCR response have been recently implicated as important triggers of antibiotic tolerance [1,71]. Experimental inactivation of *ccpA* has been shown to decrease tolerance in various clinically relevant species [57]. In *E. coli*, CCR knockout increases sensitivity to penicillin due to ablation of metabolic flux [71]. In *Streptococcus gordonii*, *ccpA* knockout ablates tolerance to multiple drug classes, both in vitro and in a rat endocarditis model [70]. Complementation with a functional *ccpA* copy is experimentally sufficient to restore tolerance in both clinical and laboratory strains [70]. *Streptococcus suis*, a zoonotic pig pathogen, and *Streptococcus pneumoniae* lose tolerance to β-lactam antibiotics when *ccpA* is mutated or deleted [70]. In methicillin-resistant *S. aureus* (MRSA), *ccpA* deletion results in severe reductions in β-lactam and glycopeptide resistance amongst highly resistant strains despite the presence of genetically-encoded resistance determinants [70,72].

*Staphylococcus epidermidis* growth and tolerance is enhanced in vitro through *ccpA*. Recently, TCA cycle activity and CCR linkage have been identified as the connecting mechanisms [73]. Interestingly, this linkage seems conserved across *Staphylococci*. In *S. aureus*, *ccpA* represses TCA cycle genes, removing inhibition of intercellular adhesion and biofilm formation, which themselves have been implicated in increased antibiotic tolerance [73]. In a clinical context, many *Staphylococcal* infections cause abscess formation, where preferred carbon sources are limited [74]. Thus, catabolism of secondary carbon sources must be highly regulated in order to adopt an antibiotic-tolerant biofilm lifestyle. As previously discussed, the formation of these tolerant biofilms has the potential to increase persister formation. Clinically, the connection between CCR, tolerance, and persistence has many implications, particularly for hosts with metabolic disorders. In hyperglycemic patients, for example, it is possible that increased glucose bioavailability triggers CCR activity, protecting pathogenic microbes from therapeutic regimens while increasing virulence and infection burden.

## 6. Sugar Metabolism and the Eradication of Persisters

Within carbon catabolism, sugar metabolism has been shown to be of particular importance in persister development. For this reason, several studies have explored the therapeutic potential of exploiting bacterial sugar metabolism to increase the efficacy of existing antibiotics against persisters [75,76,77]. It has long been known that uptake of aminoglycoside antibiotics is driven by proton motive force (PMF) [78,79]. PMF is known to be significantly lower in metabolically quiescent persister cells, which significantly limits the uptake and effectiveness of aminoglycosides [75,77,78,79]. A study by Allison et al. demonstrated that supplementation with pyruvate or metabolites that enter upper glycolysis (namely glucose, mannitol, and fructose) increased PMF and the uptake of aminoglycosides in *S. aureus* and *E. coli* [77]. As a result, they found that supplementation with these metabolites increased killing of persisters by three orders of magnitude. Conversely, metabolites that enter in lower glycolysis or the pentose phosphate and Entner–Doudoroff pathways showed little potentiation. Additionally, mannitol and fructose increased the efficacy of gentamicin against biofilms in vitro and in vivo by 4 and 1.5 orders of magnitude, respectively. However, the same potentiating effect was not observed with β-lactams. Because β-lactams require active bacterial growth for efficacy, this finding demonstrates that the persister cells have not been induced into an actively growing state by the addition of the metabolites. Additionally, treatment with the protonophore carbonyl cyanide m-chlorophenyl hydrazine (CCCP), an uncoupler of oxidative phosphorylation that reduces PMF, abolished the potentiating effect seen with aminoglycoside treatment. Taken together, these findings suggest that supplementation with central carbon metabolites induces PMF and facilitates uptake of aminoglycosides, thus potentiating their efficacy against persisters [66,77].

Similar work has been recently published using *P. aeruginosa* [75,76]. In this case, metabolites from the lower TCA cycle and glycolysis, namely fumarate, succinate, pyruvate, and acetate, sensitized persister and biofilm cells to the aminoglycoside tobramycin [75]. Conversely, supplementation with the upper TCA cycle metabolite glyoxylate was found to have a protective effect. As demonstrated by Allison et al., these effects appear to be largely explained by the changes within central carbon metabolism [75,77]. Supplementation with fumarate stimulated the TCA cycle and electron transport chain activity, thus generating PMF and facilitating uptake of tobramycin. Conversely, glyoxylate decreased cellular respiration while having no significant impact on PMF. Interestingly, supplementing both fumarate and glyoxylate increases PMF and aminoglycoside uptake while decreasing respiration. These cells remain tolerant to aminoglycosides, indicating that decreased cellular respiration can reduce toxicity and compensate for increased uptake [75].

Based on these observations, it is clear that bacterial metabolism and nutrient availability, particularly of sugars and central carbon metabolites, are important determinants of antibiotic efficacy against persisters. Therefore, it is important to understand the availability of these nutrients within the host during infection and how they alter bacterial metabolism. The use of next-generation tools will undoubtedly aid in addressing multi-faceted and complex questions such as this (Figure 1A). For example, metabolomic techniques can be utilized to characterize the metabolites present within a given niche inside the host [67]. Pairing this metabolomic data with transcriptomic data from bacteria isolated from the microbiome or an infection may lend insights into the interplay between host and pathogen metabolism. Doing so may also help identify conditions within the host that are likely to foster the development of tolerance and persistence.

## 7. Cellular Permeability, Proton Motive Force, and Persistence

Carbon metabolism, particularly of sugars, has been demonstrated to have direct and indirect effects on antibiotic uptake and efflux in persistent and tolerant bacteria. Therefore, increasing efflux or decreasing membrane permeability may represent a complementary strategy to tolerate antibiotics by preventing their intracellular accumulation. For example, the uptake of aminoglycoside antibiotics has been demonstrated to be highly PMF-dependent [78,79]. PMF is known to be significantly lower in metabolically quiescent persister cells, which significantly limits the uptake and effectiveness of this particular class of antibiotics [75,77,80]. As discussed previously, stimulating PMF through supplementation with TCA cycle metabolites has been shown to increase the uptake and efficacy of this class of antibiotics against persisters [75,77]. PMF also has an indirect effect on antibiotic uptake through the action of efflux proteins. In total, there are four major families of efflux proteins found in prokaryotes that utilize PMF as an energy source: major facilitator (MF), multidrug and toxic efflux (MATE), resistance-modulation-division (RND), and small multidrug resistance (SMR) [79,81,82,83]. *P. aeruginosa* has been found to overexpress various efflux pumps that provide protection against multiple classes of antibiotics during aminoglycoside exposure or biofilm growth [84,85,86,87]. Furthermore, this response appears to be dependent on dose and length of antibiotic exposure, suggesting that these are adaptive responses [84]. Conversely, inhibiting efflux pumps in *P. aeruginosa*, *E. coli*, and *M. tuberculosis* have been found to sensitize those bacteria to various classes of antibiotics [88,89]. Within *P. aeruginosa* biofilms, the expression pattern of the MexAB-OprM efflux pump was found to be highest at the substratum, where oxygen and nutrient availability is lowest [85].

Perhaps the most compelling evidence for the role of efflux in bacterial persistence can be found in a 2016 article by Pu et al. [90]. In this work, *E. coli* persister cells were observed to have reduced levels of cytoplasmic β-lactam accumulation due to enhanced expression and activity of the central efflux component TolC [90]. Eliminating the *ompF* and *ompC* channels (which allow for diffusion of β-lactams) did not significantly alter persister formation rates or change the intracellular antibiotic concentration relative to non-persister cells. However, knocking out or inhibiting TolC significantly attenuated persister formation and increased intracellular levels of antibiotics [90,91]. It should be noted that these persister cells were confirmed to be metabolically dormant, suggesting that the persistence phenotype encompasses both passive (reduced metabolism) and active (efflux) responses to antibiotics.

Exploiting the permeability of persister cells without modulating bacterial metabolism may present an alternative strategy to treating infections [92]. Early studies using daptomycin demonstrated that it was effective in a concentration-dependent manner against stationary phase and metabolically arrested MRSA [93]. Furthermore, daptomycin was found to be significantly more effective in these situations than β-lactams, which require active bacterial growth. Daptomycin increases cellular permeability by disrupting outer bacterial membranes, thus bypassing the requirement of active metabolism to be effective [93].

Modulating cellular permeability of bacteria may also increase the efficacy and expand the spectrum of activity of existing antibiotics [94,95]. Supplementation with ionic silver has been shown to increase the membrane permeability of Gram-negative biofilm cells by stimulating production of hydroxyl radicals that disrupt disulfide bonds and result in misfolded membrane proteins [95]. This disruption of membrane permeability was found to potentiate the activity of bactericidal antibiotics —ampicillin, ofloxacin, and gentamicin—while sensitizing *E. coli* to vancomycin, a Gram-positive-specific antibiotic. Furthermore, silver was able to enhance the activity of gentamicin in a mouse biofilm infection model [95]. Silver was also observed to potentiate gentamicin in the presence of CCCP, suggesting that this effect is not PMF-dependent [94]. Taken together, these findings suggest that disrupting membrane permeability of metabolically dormant biofilm cells may be capable of expanding the spectrum of activity of current antibiotics [94,95]. Similar effects have also been observed when aminoglycosides are administered after a hypoionic shock; however, the exact mechanism and therapeutic potential of this strategy is currently unclear [83].

To date, most of the studies linking cellular permeability to antibiotic tolerance in biofilms and persisters have been performed in vitro. As a result, the clinical relevance of this phenomenon is currently unclear. However, utilization of next-generation sequencing and its integration with older technologies may enable researchers to shed light on the role that cellular permeability and drug uptake play during antibiotic treatment of an infection. For example, fluorescently labeled antibiotics have been used in numerous studies to measure cellular drug uptake in vitro [75,77,90]. It may be possible to utilize these compounds to measure drug uptake within a population of bacteria isolated from an infection model in vivo. Furthermore, their use could allow researchers to sort bacteria using fluorescence-activated cell sorting (FACS) based on their level of drug uptake and perform transcriptomic analysis on the resulting populations. Additionally, the use of animal models would enable researchers to manipulate host metabolism to determine its impact on bacterial drug uptake and efflux during infection and antibiotic therapy.

## 8. Stress Responses and Persistence: The Stringent Response

Nutrient limitation, metabolic flux, drug efflux, and growth rate are not the only mechanisms by which persisters can arise. As the PASH model states, active transcriptional responses can trigger persisters, and major transcriptional responses are undoubtedly coupled with metabolic shifts. Recently, links between CCR and antibiotic tolerance have implicated the involvement of the stringent response [96]. The stringent response is a stress response pathway that activates during amino acid deprivation, fatty acid limitation, and other stressors [97,98]. Stringent response pathways are activated via (p)ppGpp alarmone concentrations, which modulate subsequent cellular responses such as transcription, replication, and gyrase-mediated negative DNA supercoiling [28]. (p)ppGpp is synthesized and maintained by members of the RelA/SpoT Homolog (RSH) enzyme superfamily [99,100]. When cellular concentrations of (p)ppGpp are high enough, the alarmone interacts with RNA polymerase and the DskA ribosome binding protein, ultimately reducing translational fidelity due to a reduction in the pool of filled aminoacyl-tRNAs [28]. Because the stringent response can modulate so many processes involved in direct targets of antibiotics, it has been implicated in tolerance development and biofilm recalcitrance, which likely share mechanistic triggers with persister development [28].

Bacterial mutants lacking the stringent response, such as a *relQ-rsh* double knockout in *Enterococcus faecalis*, exhibit divergence in various metabolic operons, glycerol uptake, and glycerol metabolism [96]. These processes have been demonstrated to be under the control of CcpA in *E. faecalis*. This double knockout strain has significantly perturbed levels of *ccpA* transcription, indicating an inability to accurately sense metabolic cues and properly adapt. In *S. aureus*, double knockouts of *rel*-*rsh* have aberrant intracellular pools of (p)ppGpp, suggesting an inability to control the pace or directionality of carbon flux [96]. This inability to properly adapt to nutrient availability changes likely leads to nutrient starvation and limitation. This leads to dysbiosis of NAD+/NADH ratios, increased ROS generation, and unbalanced cellular homeostasis [96].

Ghosh et al. found that when *Mycobacterium smegmatis* populations were challenged with nutrient depletion, stringent response pathways were activated, representing a form of adaptive switching that generates persisters [101]. In vitro deletion of *relA* in *E. coli* ablates (p)ppGpp synthesis, and experimental nutrient starvation fails to elicit penicillin tolerance in this mutant [12]. Furthermore, the stringent response appears to be critical to persistence development in *E. coli*, as deletion of several pathway components inhibits persister formation in vitro [102]. In *P. aeruginosa*, antibiotic tolerance in nutrient-limited and biofilm contexts is mediated by active responses to starvation rather than by passive effects of growth and arrest, which closely resembles the PASH model of persistence [103]. In Pseudomonads, the stringent response can be linked to tolerance via reduction of oxidative stress in cells. By inactivating protective mechanisms, biofilms become sensitized to multiple antibiotic classes by several orders of magnitude. In experimental knockouts of *relA* and *spoT*, cells were unable to produce (p)ppGpp during serine starvation [103]. When challenged with ofloxacin during starvation, wild type cells had a 2300-fold reduced killing, while knockout strains exhibited only a 34-fold reduction in antibiotic killing [103]. Ultimately, stringent response inactivation appears to modulate antibiotic tolerance via relief of oxidant stress, and this stress response has likely conserved functionality in persister cell formation.

The stringent response has also been linked to indole-mediated antibiotic tolerance. Vega et al. found that indole production in *Salmonella enterica* increased basal tolerance and that indole signaling could be induced in both monoculture and in co-culture with *E. coli* [104]. Indole production during stationary phase coupled with nutrient limitation leads to increased levels of persisters. Indole production functions as a form of intraspecies signaling to promote transcriptional activation of efflux pumps and oxidative stress protection in neighboring cells [12]. (p)ppGpp overexpression increases antibiotic tolerance and inhibits peptidoglycan and phospholipid synthesis, indicating a link between amino acid starvation, oxidative stress, the stringent response, and antibiotic tolerance [28].

As a generalized stress response with protective functionality against nutrient limitation and oxidative stress, it seems likely that stringent response activation is co-opted for an in-host lifestyle. Bacteria frequently encounter nutrient limitation within hosts and must subvert oxidative damage from the host immune system in order to colonize, establish infection, and persist [74,105]. Additionally, bacteria must be able to withstand nutrient limitation during host-to-host transmission events. Transcriptomics could be implemented to study the role of the stringent response in in vivo persister formation during an induced infection. In a clinical setting, biopsy samples could be subjected to the same transcriptional profiling.

## 9. Stress Responses and Persistence: The SOS Response

The stringent response is not the sole stressor implicated in persistence development. Under biofilm conditions, stringent response activation increases basal expression of the SOS DNA repair regulon [97]. The SOS response is a highly conserved gene pathway that allows cells to survive genotoxic stressors, including β-lactam and fluoroquinolone antibiotics, and is well-known to be involved in persistence development in a variety of clinically relevant species [12,29].

A pivotal study by Dorr et al. challenged the previous contention that persister formation was only due to stochastic dormancy [29]. Instead, they proposed that persister cells are actively able to survive antibiotic stress via an increase in efficient drug efflux and DNA lesion repair via either transient overexpression or environmental activation of the SOS pathway [29]. Experimental knockdown of *recA* and *recBC*, key players in the SOS response, caused complete ablation of persister formation after 6 hours of antibiotic exposure, suggesting that persisters experienced and were unable to mitigate antibiotic-induced DNA lesions. *E. coli* strains that constitutively express the SOS regulon had a 20-fold increase in persister formation upon ciprofloxacin challenge. When challenged with mitomycin C, these mutants demonstrated a 180-fold increased induction of persistence, suggesting a functional SOS response is necessary for antibiotic persistence [29]. Bernier et al. expanded upon this work and found that SOS induction is necessary for ofloxacin tolerance, and proposed that SOS induction might lead to persistence development in biofilms [59]. Their findings support the complex interconnectedness between metabolic flux systems, the stringent response, and SOS repair pathways in promoting persistence development.

A major trigger for persistence development is exposure to sub-inhibitory concentrations of antibiotics, which mimics in vivo drug accumulation after clinical administration [1]. Daily dosing of aminoglycosides selects for almost complete persister enrichment in *Klebsiella pneumoniae* and periodic daptomycin exposure leads to high persister enrichment in *S. aureus* [106]. When challenged to multi-antibiotic panels, *E. coli* persister formation was enhanced by both ciprofloxacin and gentamicin treatment while *S. aureus* persistence activity increased under ampicillin treatment. These findings suggest some interspecies variation or drug-specific variation in persistence mechanisms. Interestingly, ampicillin pretreatment increased the rate of cross-tolerance to non-related drug classes due to β-lactam activation of the SOS pathway in *S. aureus* [26]. SOS-deficient *E. coli* strains failed to produce persisters during ciprofloxacin challenge but were able to produce gentamicin persisters since gentamicin does not directly cause DNA lesions [26]. This suggests that persisters are actively synthesizing DNA and are sensitive to perturbations in DNA integrity.

Sub-inhibitory drug concentrations are of high clinical relevance. Realistically, serum antibiotic levels are only at inhibitory concentrations for a short portion of the regimen [107]. As a result, bacteria spend most therapeutic time at sub-inhibitory concentrations while inside a host, and this is drastically exaggerated in biofilm antibiotic exposure. Interestingly, it is this transient concentration that elicits and selects for tolerance [108]. However, insufficient data is available regarding bacterial responses during these transient, sub-therapeutic concentrations. Significant insight can be gained by profiling physiological and transcriptional responses of pathogens isolated from the site of infection as the effective antibiotic concentration is reduced by host metabolism.

## 10. Future Directions

The past decade has brought many advancements in the study of bacterial persistence. Despite these advances, persistent infections remain a major public health burden and work is needed to translate new discoveries to improved clinical outcomes. One potential area of research that could help bridge this gap is determining the role that host metabolism plays in bacterial persistence. To date, the vast majority of persistence research has been conducted in vitro under nutrient conditions that differ considerably from what is found in vivo. Human metabolism is a complex phenotypic trait that is dependent on a multitude of factors such as genetics, diet, and microbiome composition [109,110,111,112]. Further complicating the role of human metabolism is the fact that the human host comprises of a multitude of micro-niches that harbor vastly different nutrient conditions. A breadth of research has demonstrated that metabolic activity is a key factor in the development of bacterial persistence. Therefore, it is likely that nutrient availability in these micro-niches may act as a determinant of bacterial metabolism and thus persister formation. If so, understanding the impact that human metabolism plays in bacterial persistence and treatment efficacy is crucial to improving patient outcomes. Furthermore, uncovering the links between host metabolism and bacterial persistence could open the door to new therapeutic strategies that improve the efficacy of treatment by modulating host metabolism. Such strategies could lay the foundation for personalized medicine by allowing medical professionals to tailor treatment based on infection site and the patient’s overall metabolic state.

Another untapped area of research is the potential link between persister formation and the microbiome. Most, if not all, pre-existing persister research has been conducted in vitro using human pathogens. However, it is unknown if persister formation occurs within the complex polymicrobial communities that comprise the microbiome. Though persistence is typically viewed negatively in the context of recurrent clinical infections, it is possible that it may serve a beneficial role in the context of the microbiome. Antibiotic treatment is known to decrease the diversity and count of bacteria in a number of niches within these communities, which, in turn are associated with dysbiosis and other negative health outcomes [113,114,115,116,117,118]. However, a form of persistence may enable beneficial microbes to survive perturbations such as antibiotic treatment, infection, or dietary shifts, thus allowing them to replenish a healthy microbiota. Conversely, these strategies could also explain the bloom of opportunistic pathogens following antibiotic therapy. In either case, it is crucial to understand if and how persistence mechanisms are utilized in the context of the microbiome.

Addressing these questions would have been logistically daunting in past decades due to the diversity of the microbiome and the inability to culture many of its resident microbes. However, the advance of next-generation sequencing technologies in the past decade has enabled new insights into the development of persistence. For example, experiments utilizing RNA-Seq demonstrated that persisters overexpress the TolC efflux pump, indicating a previously unknown role of drug efflux in this phenomenon [90]. Additionally, Henry et al. recently developed a platform that integrated fluorescence-activated cell sorting (FACS), traditional antibiotic susceptibility assays, and next-generation sequencing to assay persister physiology [41]. The extreme rarity of persisters within polymicrobial communities makes many in vivo analyses logistically difficult. The development of single-cell sequencing technologies, combined with persister enrichment protocols such as FACS and LCMD, present robust avenues for analysis in vitro and in vivo [42,43,44,45,46,47,48]. Critically, many of the methods that utilize these technologies are culture-independent. Therefore, they may serve as powerful tools that will allow researchers to determine the mechanisms underlying persistence in complex microbial communities or during infection.
